# Modeling *Salmonella* Typhimurium Inactivation in Dry-Fermented Sausages: Previous Habituation in the Food Matrix Undermines UV-C Decontamination Efficacy

**DOI:** 10.3389/fmicb.2020.00591

**Published:** 2020-04-08

**Authors:** Yhan S. Mutz, Denes K. A. Rosario, Patricia C. Bernardes, Vania M. F. Paschoalin, Carlos A. Conte-Junior

**Affiliations:** ^1^Post Graduate Program in Food Science, Institute of Chemistry, Federal University of Rio de Janeiro, Rio de Janeiro, Brazil; ^2^Analytical and Molecular Laboratory Center, Faculty of Veterinary Medicine, Fluminense Federal University, Niterói, Brazil; ^3^Center for Food Analysis, Technological Development Support Laboratory (LADETEC), Federal University of Rio de Janeiro, Rio de Janeiro, Brazil; ^4^Department of Food Engineering, Federal University of Espirito Santo, Alto Universitário, Alegre, Brazil; ^5^National Institute of Health Quality Control, Oswaldo Cruz Foundation, Rio de Janeiro, Brazil

**Keywords:** inactivation kinetics, Weibull model, dry-cured meat, non-thermal technology, food safety, preservation of sensory characteristics

## Abstract

The effects of previous *Salmonella* Typhimurium habituation to an Italian-style salami concerning pathogen resistance against ultraviolet-C light (UV-C) treatment were modeled in order to establish treatment feasibility for the decontamination of dry-fermented sausage. *S.* Typhimurium following 24 h habituation in fermented sausage (habituated cells) or non-habituation (non-habituated cells) were exposed to increasing UV-C radiation treatment times. The Weibull model was the best fit for describing *S.* Typhimurium UV-C inactivation. Heterogeneity in UV-C treatment susceptibilities within the *S.* Typhimurium population was observed, revealing intrinsic persistence in a sub-population. UV-C radiation up to 1.50 J/cm^2^ was a feasible treatment for dry-fermented sausage decontamination, as the matrices retained instrumental color and lipid oxidation physiochemical characteristics. However, habituation in the sausage matrix led to a 14-fold increase in the UV-C dose required to achieve the first logarithm reduction (δ value) in *S.* Typhimurium population. The results indicate that, although UV-C radiation might be considered an efficient method for dry-fermented sausage decontamination, effective doses should be reconsidered in order to reach desirable food safety parameters while preserving matrix quality.

## Introduction

Ready-to-eat meat products may pose a safety risk for consumers, due to contamination by foodborne pathogens, such as *Salmonella*, during either the pre or post-processing stages (Mutz et al., 2019, 2020). *Salmonella enterica* is a foodborne pathogen able to contaminate a wide range of foods from both vegetal and animal origins, raising public health authority concerns worldwide (Ferrari et al., 2019). Salmonellosis is the second most reported cause of gastrointestinal infection in humans, accounting for 29% of total foodborne outbreaks in United States and 30.7% in the European Union (Center for Disease Control and Prevention [CDC], 2019; European Food Safety Authority [EFSA] and European Centre for Disease Prevention, and Control [ECDC], 2019). *Salmonella* Typhimurium is the most frequent serovar concerning animal-based food contamination (Ferrari et al., 2019) and the second serovar involved in outbreaks (Gossner et al., 2012; Scavia et al., 2013; Andreoli et al., 2017).

Physicochemical matrix characteristics such as low water activity, a moderately acidic pH and indigenous competitive microbial population inherent to dry-fermented sausages are natural hurdles for microbial growth (Leistner and Gorris, 1995). The harmful combination effects of these sublethal stresses on bacterial cells is the pillar to guarantee the safety of dry-cured meat products. However, even traditional manufacturing process, such as those well established and used for centuries in the preparation of Italian salami, a dry-fermented sausage, cannot ensure the absence of *Salmonella* spp. contamination (Bonardi et al., 2017). As no specific step is included along the manufacturing process of dry-fermented meat products for pathogen elimination, the abiotic stresses exerted by the food matrix can transform pre-existent foodborne pathogens into more persistent and even more virulent organisms (Mutz et al., 2020).

Several studies on different non-thermal alternative technologies able to kill pathogens and maintain the safety of dry-fermented sausages have been performed (Cabeza et al., 2009; Chun et al., 2009; Porto-Fett et al., 2010; Ganan et al., 2013; Rosario et al., 2019). The use of non-thermal technologies to guarantee the safety of dry-fermented sausages has been addressed, since high temperatures may lead to detrimental effects on the sensory characteristics of these meat products. Among the different non-thermal technologies, ultraviolet-C light (UV-C) is noteworthy, as it is considered environmental-friendly, displaying low costs and being easily applied to the food industry (Koutchma, 2009). UV-C treatment acts at a superficial level, limiting the practical application of this technology to sanitize meat products. However, this is not a drawback for the decontamination of sliced ready-to-eat products, since post-processing contamination occurs mostly at the product surface, during handling, slicing, and packing, and UV-C may be applied on both sides of the slices to guarantee efficient sanitization (Ganan et al., 2013). Recently, several food matrix characteristics that may impact the efficacy of UV-C microorganism inactivation have been emphasized (Gayán et al., 2011; Geveke et al., 2011; Canto et al., 2018; Monteiro et al., 2018; Castro et al., 2019). However, matrix habituation effects exerted on foodborne pathogens is a novel aspect which has not yet been adequately addressed.

In this context, the aim of the present study was to evaluate the efficiency of UV-C light on the inactivation of *Salmonella* Typhimurium habituated to the stress conditions that take place during pathogen long-term exposure to the dry-fermented sausage matrix. The feasibility of UV-C light application on the decontamination of dry-fermented sausages was also addressed by evaluating sausage quality following *Salmonella* inactivation treatment.

## Materials and Methods

### Dry-Fermented Sausages

Dry-fermented sausages, a type of salami, were purchased at a local market in the municipality of Niterói, RJ (Brazil) and stored under refrigeration following manufacturer recommendations until analysis. The declared sausage composition was as follows: pork meat, skin milk powder, fat (lard), salt, white wine, sugar, mix of spices (mostly black pepper), sodium ascorbate, and sodium nitrate, containing an average 3.8% of carbohydrates, 25% of proteins and 32% fat. Samples presented an average pH of 5.4 ± 0.2 and a_w_ of 0.85 ± 0.1. Sausages were cut into 1.0 mm thick slices using a meat slicer (Arbel^®^ Ftd 178 MC/MC-X 3.0, SP, Brazil) using blades previously disinfected with 70% alcohol and rinsed with sterile distilled water.

### Bacterial Culture Conditions

S*almonella enterica* serovar Typhimurium ATCC 14028 stock cultures were maintained in brain heart infusion broth (BHI) (BD^®^, NJ, United States) containing 20% (v/v) glycerol at – 80 ± 1°C. Cultures were also grown at 4°C in Hektoen enteric agar (HE) (Liofilchem^®^, Teramo, Italy) and renewed monthly. Working cultures were obtained by transferring one single colony displaying a characteristic morphology from the HE agar to 10 mL of BHI broth. Cells were cultured overnight at 37 ± 1°C until reaching the late stationary growth phase, at a cellular density of approximately 10^8^ CFU/mL, confirmed by HE agar counts. Plating was performed using a Spiral Plater Eddy Jet 2 (IUL Instruments, Barcelona, ESP) and enumeration was carried out using an electronic Flash & go counter (IUL instruments).

### Exogenous Sausage Contamination and *Salmonella* Typhimurium Habituation to the Dry-Fermented Sausage Matrix

Sliced-sausage samples were set as individual 10 g portions in polyethylene bags for exogenous contamination with *Salmonella* Typhimurium. Cultures were centrifuged in a Sorvall ST 16 centrifuge (Thermo Fisher, GER) at 5,580 × *g* for 10 min at 4°C. The cell pellets were resuspended in 0.1% casein peptone (Sigma-Aldrich^®^, Germany), in order to obtain two different inoculant levels, 10^8^ CFU/ml and 10^6^ CFU/ml. Concerning the 10^8^ CFU/ml inoculum, cells from the overnight culture were suspended in 2 mL of the peptone solution. For the 10^6^ CFU/ml inoculum, the harvest cells from the overnight culture were suspended in 10 mL of a peptone solution and were serially diluted. When the proper dilution was achieved, the cells were harvested by centrifugation and then resuspended in 2 mL of fresh peptone solution. Two distinct inocula were used to achieve the same cell concentration on the sausages prior to UV-C treatment, due to the extent of pathogen inactivation caused by the 24 h habituation on the fermented-sausage matrix.

Cell habituation was performed by exposing *S*. Typhimurium cells to sausage matrices for 24 h at 25 ± 1°C prior to the UV-C treatment, an experimental condition already proven to induce *S.* Typhimurium resistance against simulated gastric fluid, confirming the enhancement of bacteria cells to adverse physical conditions (Mutz et al., 2019).

A 100 μL aliquot of the cell suspensions, corresponding to 10^8^ CFU/ml of *S.* Typhimurium in samples to be subjected to prior sausage habituation and 10^6^ CFU/ml in samples not subjected to habituation, were spot-inoculated and spread on each side of the sausage slices using a sterile, bent glass rod. The exogenously contaminated sliced sausages were then air-dried in a laminar flow and vacuum-sealed (AP450 vacuum sealer, TECMAQ, Brazil). Sliced samples that did not undergo the habituation period were determined to be at 4.28 ± 0.17 log CFU/g, while samples subjected to a 24 h habituation period were determined to be at 4.10 ± 0.07 CFU/g. Student’s *T* test applied to the initial counts showed no difference in number of cells between both inocula (*p* < 0.05).

The absence of *Salmonella* spp. in dry-fermented sausage samples prior to exogenous contamination was confirmed by HE agar plating.

### Application of UV-C Light

The UV-C light emitting equipment comprised six 30 watts UV-C lamps interposed by six 55 Wwatt UV-C lamps (OSRAM HNS, OFR, Munich, Deutschland), as designed by Lazaro et al. (2014). Lamps were warmed-up prior to treatments to stabilize UV-C radiation intensity (1.53 ± 0.07 mW/cm^2^), monitored by a MRUR-203 UV radiometer (Instrutherm Instrumentos de Medição Ltda, SP, Brazil) placed inside the equipment, where the detector was sealed in the same vacuum package used for the sausage slices. Individual sausage slice packages were placed in the center of the UV-C equipment, 14 cm distant from the UV-C lamps. The sausage slices were exposed to UV-C radiation at increasing time periods (0, 0.3, 0.8, 1.1, 3.3, 5.7, 12.4, and 17.0 min), set to achieve 0.03, 0.10, 0.30, 0.50, 1.10 to 1.50 J/cm^2^ doses. Doses were calculated as E = I × t, where E is the dose (energy density) in J/cm^2^, I is intensity in mW/cm^2^ and t is the time in min. UV-C lamp intensities were monitored every 5 s until each of the seven desired doses were achieved.

### *Salmonella* Typhimurium Enumeration

After each treatment, 10 g of the treated samples were aseptically collected and homogenized in a digital stomacher (YK Tecnologia, RS, Brazil) containing 90 mL of 0.1% peptone solution. Decimal serial dilutions of the homogenate ranging from 10- to 1000-fold were plated on HE agar plates and incubated at 37 ± 1°C for 24–48 h in order to estimate cell survival, expressed as log CFU/g.

### *Salmonella* Typhimurium Inactivation Kinetics

To describe the UV-C inactivation kinetics of *S.* Typhimurium habituated to dry-fermented sausage matrices, data following the UV-C treatments were used to fit three distinct survival models. Models were adjusted by the GInaFIT version 1.6 software (Katholieke Universiteit Leuven, Belgium), a Microsoft^®^ Excel freeware (Geeraerd et al., 2005).

(1) Log-Linear Bigelow (Bigelow and Esty, 1920):

(1) $$log_{10}\left(N_{t}\right)=log_{10}\left(N_{0}\right)-\left(\frac{t}{D}\right)$$

where *N*_0_ is the inoculum (log CFU/g), *N*_*t*_ is the number of survivals cells (log CFU/g) at the time *t* (min), and *D* is the decimal reduction time, which is the time under UV-C treatment required to obtain a 1 log_10_ population reduction and t is the UV-C dose.

(2) Geeraerd-tail model (Geeraerd et al., 2000)

(2) $$\begin{array}{c} log_{10}\left(N_{t}\right)=log_{10}\left(10^{log_{10}\left(N\left(0\right)\right)}-10^{log10\left(N_{res}\right)}\right)\cdot \\ \;\exp e^{-k_{max}t}+10^{log_{10}\left(N_{res}\right)} \end{array}$$

where *N*_0_, *N*_(_*_*t*_*_)_ and *t* are the same as described above, *N*_*res*_ is the UV-C resistant population and *k*_*max*__*T*_ is the maximum specific inactivation dose (J/cm^2^)^–1^.

(3) Weibull model (Mafart et al., 2002)

(3) $$log_{10}\left(N_{t}\right)=log_{10}\left(N_{0}\right)-{\left(\frac{t}{\delta }\right)}^{p}$$

where *N*_0_, *N_(t__)_* and *t* are the same as described above, δ is the time required for the first Log_10_ population reduction and *p* is a shape parameter, where *p* > 1 indicates an upward concavity and *p* < 1, a downward concavity.

The goodness-of-fit of the tested models was assessed through the adjusted coefficient of determination (*R*^2^_*adj*_) and mean square error (MSE).

### Physicochemical Analyses

Instrumental color parameters were determined according to the CIE color scale using a portable CM-600D spectrophotometer (Konica Minolta Sensing Inc., Osaka, Japan) equipped with an illuminant A, 8 mm aperture, and 10° standard observer (Monteiro et al., 2017). Lightness (*L*^∗^) and redness (*a*^∗^) were determined, and the total color difference (ΔE) between dry-fermented sausages treated by UV-C light and non-treated sausages was calculated according to Silveira et al. (2018). In addition, surface reflectance at 650/570 nm was measured to estimate cured meat fading (American Meat Science Association [AMSA], 2012). All data were obtained using the Spectra Magic NX version 2.70 software (Konica Minolta Inc.).

Lipid oxidation was evaluated by the thiobarbituric acid-reactive substance (TBARS) method described by Yin et al. (1993) with modifications (Joseph et al., 2012). Absorbances at 532 nm were determined using a UV-1800 spectrophotometer (Shimadzu Co., Kyoto, Japan). Results were presented as mg malondialdehyde/kg of dry-cured meat according to a standard curve.

### Statistical Analyses

All microbiological and physicochemical analyses were conducted by assessing three independent biological replicates, followed by analytical duplicates. Data concerning UV-C radiation effects on sausage physicochemical properties were evaluated by a stepwise regression analysis using the XLSTAT software, version 2019.1.1 (Addinsoft^®^). The *F*-test was used to check model adequacy and parameter significance (*p* < 0.05).

## Results

### Model Goodness-of-Fit Assessments and *Salmonella* Typhimurium UV-C Inactivation Aspects

Model performance parameters are presented in Table 1. The variance explained by the models ranged from 0.73 to 0.98. The Weibull model displayed the higher *R*^2^_*adj*_ and the lowest MSE values for the habituated and non-habituated inactivation. Taken together, the overall evaluation indicates that the Weibull is the best model to describe *S.* Typhimurium UV-C inactivation of both habituated and non-habituated cells, thus being adopted herein.

**TABLE 1:** Statistical goodness-of-fit and Weibull model parameters for UV-C *Salmonella* Typhimurium inactivation in dry-fermented sausages.

*Salmonella* cells *status*	Model	δ*	p*	*R*^2^_*adj*_	MSE
Non- habituated	Log-Linear	–	–	0.79	0.100
	Log-Linear with tail	–	–	0.83	0.080
	Weibull	2.47 ± 1.10	0.36 ± 0.06	0.96	0.019
Habituated	Log-Linear	–	–	0.73	0.020
	Log-Linear with tail	–	–	0.84	0.011
	Weibull	34.48 ± 5.78	0.28 ± 0.03	0.98	0.001

* Weibull model parameters; δ is the time required for the first log population reduction; p is a shape parameter, where p > 1 indicates an upward concavity and p < 1, a downward concavity; R^2^_adj_ is the adjusted coefficient of determination; MSE is the mean squared error. Habituated cells were exposed for 24 h to the dry-fermented sausage matrix. Data were obtained from three independent biological replicates, followed by analytical duplicates.

**FIGURE 1:**
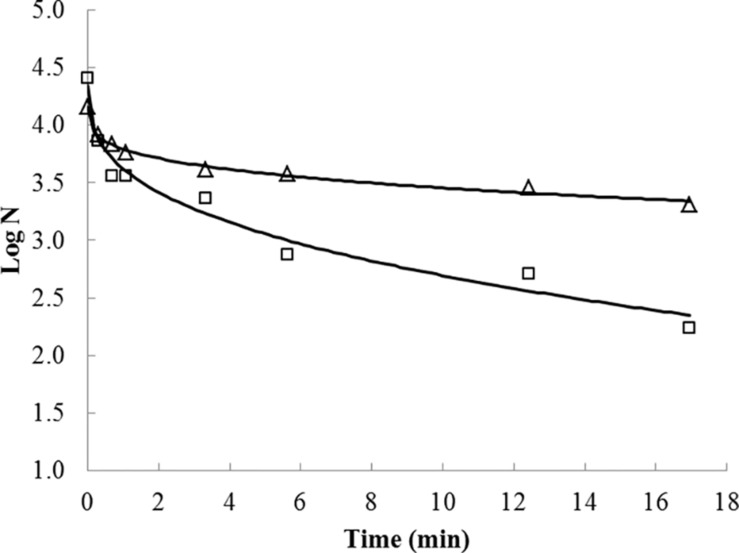
UV-C treatment effects on *Salmonella* Typhimurium inactivation on dry-fermented sausages. Fitting of the Weibull model (lines) to the observed *S.* Typhimurium inactivation data corresponding to non-habituated (squares) and habituated (triangles) cells exposed to UV-C radiation treatments.

Inactivation curves for habituated and non-habituated cells presented a positive parameter *p* shape, indicating upward concavities for the inactivation curves (Figure 1 and Table 1). A reduced inactivation rate, tending toward an asymptote, also known as the inactivation tail, was also observed toward the longest UV-C radiation exposure times in both habituated and non- habituated cells.

### Effects of Dry-Fermented Sausage Habituation on *Salmonella* Typhimurium UV-C Resistance

As previously pointed out, the survival curves were constructed by counting the survival cells in each treatment time up to 16.9 min, converted to doses of up to 15 (J/cm^2^). The UV-C treatment time required to achieve the first decimal logarithm reduction (δ value) of habituated *Salmonella* Typhimurium cells was, on average, 14-fold higher than non-habituated cells (Table 1), indicating lower UV-C lethality after the habituation treatment.

### Instrumental Color of UV-C Treated Sausages and Lipid Oxidation

Color parameters, *L*^∗^ (lightness), *a*^∗^ (redness), cured meat fading (R650/570) and the total color difference (ΔE) between treated and non-treated sausage slices are displayed in Table 2. UV-C radiation caused sausage lightness values to increase with the treatment time, with the first difference observed at the 5.7 min treatment (Table 2). Redness values presented an overall decrease, and it is noteworthy that *a*^∗^ values began to differ from the control at the lowest time treatment (Table 2). Accompanying redness, the 650/570 nm ratio was found to decrease with increasing UV-C treatments, indicating loss of cured-meat color. Total color changes (ΔE) were found to increase following UV-C treatment. Malonaldehyde (MDA) contents in sausage samples progressively increased after each UV-C treatment, reaching a maximum concentration of 1.75 mg/kg after 16.9 min treatment (Table 2).

**TABLE 2:** Instrumental color parameters and lipid oxidation estimated in dry-fermented sausages treated by UV-C radiation.

Treatment time	L*	a*	ΔE	TBARS
0.0	45.26^c,d^	11.41^a^	–	0.23^h^
0.3	45.39^c,d^	10.53^b^	0.84^d^	0.29^g^
0.7	45.09^d^	10.76^b^	1.64^b,c^	0.33^f^
1.1	45.51^c,d^	10.90^b^	1.49^c^	0.38^e^
3.3	44.99^d^	9.27^d^	2.07^b^	0.43^d^
5.7	45.84^c^	10.07^c^	2.15^b^	0.51^c^
12.4	47.05^b^	9.13^d^	2.06^b^	0.97^b^
17.0	48.29^a^	8.24^e^	3.93^a^	1.75^a^

L* luminosity; a* redness; ΔE total color difference when compared to untreated samples; TBARS is expressed as mg of MDA/kg; Different letters in the same column indicate differences (p < 0.05). Results were obtained from three independent replicates followed by analytical duplicates and are expressed as means ± standard deviation (n = 6).

## Discussion

### Model Goodness-of-Fit Assessments and *Salmonella* Typhimurium UV-C Inactivation Aspects

Ultraviolet-C light microbial inactivation is attributed to direct damage at the DNA level by the formation of mutagenic products, mostly cyclobutane-pyrimidine dimers, pyrimidine 6-4 pyrimidone photoproducts, and their Dewar isomers (Yajima et al., 1995; Bucur et al., 2018). Furthermore, the oxidative effect of UV-C on unsaturated fatty acids from lipids in the food matrix and on microorganism phospholipid bilayer cell membranes lead to the formation of free radicals, which may damage several macromolecules, such as lipids, proteins, and DNA (Rastogi et al., 2010). The extent of DNA damage by UV-C light is proportional to the amount of light exposure and the efficiency of cell repair mechanisms. However, the overall effect does not necessarily imply in a linear relationship between dose and inactivation, as noted in the death curves reported herein.

Several decontamination methods studies have reported deviations from a first order kinetic of death, including the tails commonly found in UV-C inactivation curves (Coroller et al., 2006; Chun et al., 2009; Souza and Fernández, 2011; Condón-abanto et al., 2016). The Weibull model better described the survival of habituated and non-habituated *S.* Typhimurium cells, as expected due to the concave survival curves observed herein present study. The Weibull model presented the highest *R*^2^ and the lowest MSE, indicating lower discrepancy between experimental and fitted values among the models tested herein (Granato et al., 2014).

One of the postulated hypotheses regarding the inactivation curve tailing effect is based on the heterogeneity of the cell population (Cerf, 1977), which implicates in an intrinsic dissimilarity in the resistance of each single cell against abiotic stress conditions and conservation technologies (Aspridou and Koutsoumanis, 2015). Furthermore, Coroller et al. (2006) pointed out subgroupings within the microbial population, translating this dissimilarity into resistant and susceptible sub-populations (Coroller et al., 2006). Applying the aforementioned hypotheses to the data reported herein, it is possible to infer that the sharp inactivation kinetic observed following short UV-C treatment can be considered as the death of the susceptible *S.* Typhimurium sub-population. On the other hand, the tail found during longer treatments may represent an UV-C treatment under-effect on the resistant sub-population.

An alternative hypothesis for the tailing effect in microorganism inactivation attributes the non-linearity of the inactivation curves to treatment failure in reaching target microorganisms within the food matrix (Cerf, 1977). This, in turn, can be attributed to the low penetration power of UV-C radiation (Koutchma, 2009) and its inactivation effectiveness dependence on treatment parameters and food surface characteristics (Gayán et al., 2011). Both hypotheses should be taken into account concerning the observed inactivation tail, since the variable UV-C sensitivity of subpopulations reflected as non-regular effectiveness through food matrices surfaces must be managed in order to achieve meat product safety and preservation.

### Effects of Dry-Fermented Sausage Habituation on *Salmonella* Typhimurium UV-C Resistance

Chun et al. (2009) reported a 2 log CFU/g reduction in non-habituated *S.* Typhimurium cells in ready-to-eat ham after exposure to UV-C light at 0.8 J/cm^2^, a higher inactivation than described herein. The aforementioned study detected higher *S.* Typhimurium inactivation in the matrix, however, it should be noted that the bacteria was not subjected to any matrix habituation. It should be emphasized that the efficiency of UV-C against non-habituated cells can be an overestimation of the real efficiency of the decontamination treatment.

In the present study, habituated *S.* Typhimurium cells were 14-fold more resistant against UV-C treatment than non-habituated cells. This implies in the fact that *S.* Typhimurium pre-exposed to the sausage matrix and its environmental stressful conditions are less sensitive to UV-C treatment, impacting UV-C decontamination efficacy.

The results described herein can be attributed to at least two hypotheses. First, the sub-lethal stresses caused by the meat matrix inherent physiochemical characteristics, such as low water activity and moderate acidic pH, may trigger a general cell stress response through alternative sigma factors, RpoE (σ^E^) and RpoS (σ^S^), leading to stress cross-protection and acquired tolerance against distinct stresses (Mutz et al., 2020). Indeed, increased UV-C tolerance in *S.* Typhimurium cells previously adapted to abiotic stresses have been reported (Gruzdev et al., 2011; Estilo and Gabriel, 2017). High osmolarity is known to increase σ^E^ levels, and the regulon of this general sigma factor also responds to envelope, and oxidative stresses (McMeechan et al., 2007). σ^S^, also induced by low osmolarity and/or acid stresses, is known to regulate set of enzymes involved in oxidative damage protections and DNA repair (such as catalases and exonucleases) (Eisenstark et al., 1996). Together, these pre-adaptations can elicit a more efficient response against the damage provoked by the UV-C treatment. Corroborating the hypothesis that habituated cells may acquire resistance against UV-C, Bucheli-Witschel et al. (2010) observed that *E. coli* mutants lacking *RpoS*, are more susceptible to UV-C radiation than wild-type strains (Bucheli-Witschel et al., 2010). There is, however, still a need to elucidate the molecular mechanisms behind the relationship exerted by abiotic stresses inherent to fermented sausages characteristics and the resulting UV-C resistance. Further research on gene expression by meta-transcriptomics, similar to those reported in studies dealing with other abiotic stressors (Gruzdev et al., 2012; Jia et al., 2017), and even for pulsed UV-light exposure (in *Listeria monocytogenes*) (Uesugi et al., 2016) are encouraged, in order to understand the metabolic pathways and cellular processes impacted by UV-C in *S*. Typhimurium.

Secondly, pathogen internalization into the sausage matrix should also be taken into account (Lim and Harrison, 2016), since the random penetration of bacteria in the dry-cured matrix is known to play a role in impairing UV-C treatment effectiveness (likewise for pulsed UV light) (Rajkovic et al., 2017). Taken together, these two possibilities may lead to the inference that, in order to estimate the real effectiveness of the UV-C decontamination treatment, the effects of pathogen habituation to the stressful conditions within the dry-cured meat matrix should be assessed, in order to mimic the adverse scenario which foodborne pathogens undergo in dry-fermented meat matrices.

### Instrumental Color and Lipid Oxidation

Color is the first quality attribute perceived by consumers when evaluating meat products, used as a synonym for their quality, directly influencing purchase intentions and, therefore, of the utmost importance. Lightness increases in UV-C treated meat are usually associated to protein denaturation, as myoglobin content is inversely related to *L*^∗^ values (Canto et al., 2016, 2018; Cunha et al., 2018). Furthermore, UV-C can also induce the denaturation of sarcoplasmic and myofibrillar proteins, leading to exposure of hydrophobic groups and increased free water content, altering meat surface reflectance (Canto et al., 2018). Decreased *a*^∗^ values are correlated with the heme pigment oxidation process. UV-C light is a well-known oxidizing agent, therefore displaying the ability to affect meat color, as changes in protein conformation or in redox state alters the hemo-chrome of this matrix (Sun et al., 2009). Nytrosil-hemochrome present in cured meats, similar to myoglobin in fresh ones, is sensitive to light exposure and oxidative reactions, which leads to fading in cured products (Suman and Joseph, 2013).

The collective increase in lightness and decrease in meat redness are responsible for dry-cured meat discoloration (Wambura and Verghese, 2011; Ganan et al., 2013). The cured-meat discoloration process is reinforced by the absorbance ratio 650/570 nm that describes cured-meat fading, which decreases with UV-C treatment (American Meat Science Association [AMSA], 2012). The total color changes observed in UV-C treated sausages followed an increasing trend for redness (*a*^∗^) and lightness (*L*^∗^) with increasing UV-C treatment time/intensity (Table 2). In order to obtain a tangible estimation of the extent of color changes (ΔE), an ΔE value of 5 units can be adopted as the threshold for easy food color change perception (Tazrart et al., 2016; do Rosário et al., 2017; Canto et al., 2018). Thus, applying the aforementioned reference to ΔE data, although the total color difference in sausage slices increased, no perceptive change should be observed.

Another undesirable effect of UV-C treatment is lipid oxidation, as it results in loss of nutritional value and the development of a rancid off-flavor perceived by consumers when the amount of MDA in treated-meat reaches a threshold of 2 mg of MDA/kg meat (Campo et al., 2006). UV-C radiation is an oxidant agent that promotes free radical formation, increasing lipid oxidation even in low-moisture meat matrices (Barden and Decker, 2013; Monteiro et al., 2018). The MDA content observed herein increased throughout the UV-C treatment period, corroborating the observed color changes in the same dose range. This simultaneous change in TBARS and color parameters may result from meat pigment oxidation, as the reactive intermediates formed by the UV-C treatment are capable of enhancing lipid oxidation (Faustman et al., 2010). However, it is noteworthy to mention that the sausage samples did not reach the aforementioned MDA limit, even at the highest treatment time, which reinforces no harmful effect on product acceptance.

## Conclusion

The Weibull model fitted UV-C *Salmonella* Typhimurium inactivation behavior. The inactivation curve presented an upward concavity following by an inactivation tail toward high treatment time/intensity, indicating a natural heterogeneity within the *S.* Typhimurium population toward UV-C susceptibility. Habituation of *S.* Typhimurium cells to the fermented sausage matrix lead to a 14-fold increase in pathogen resistance against UV-C treatment. This undermined UV-C efficacy has practical consequences, as the establishment of effective doses based on non-habituated cells will lead to UV-C treatment efficacy overestimations. The physicochemical changes in dry-fermented sausages in the experimental conditions did not point to major product quality deteriorations. However, increases in treatment time/intensity to meet the inactivation of one logarithm decimal reduction of habituated *S.* Typhimurium, herein estimated at 34 min, may compromise food quality and its acceptance.

The approach of treatment efficacy assessments using habituated pathogens is encouraged in order to evaluate the UV-C decontamination methodology in a more critical perspective concerning decontamination effectiveness and food quality preservation and acceptance. Furthermore, future meta-transcriptomics studies are encouraged, to unveil the cellular mechanisms behind the relationship between stress responses and increased UV-C resistance.

## Data Availability Statement

The datasets generated for this study are available on request to the corresponding author.

## Author Contributions

YM and DR contributed to conceptualization, methodology, validation, formal analysis, investigation, data curation, and original draft preparation. PB, CC-J, and VP were responsible for the resources, reviewing and editing the manuscript. CC-J and VP supervised the study. CC-J was responsible for the funding acquisition.

## Conflict of Interest

The authors declare that the research was conducted in the absence of any commercial or financial relationships that could be construed as a potential conflict of interest.
